# Accumulation of Heavy Metals in Rice (*Oryza sativa*. L) Grains Cultivated in Three Major Industrial Areas of Bangladesh

**DOI:** 10.1155/2022/1836597

**Published:** 2022-03-08

**Authors:** G. M. M. Anwarul Hasan, Anuj Kumer Das, Mohammed A. Satter

**Affiliations:** ^1^Institute of Food Science and Technology, Bangladesh Council of Scientific and Industrial Research, Dr. Qudrat-I-Khuda Road, Dhanmondi, Dhaka-1205, Bangladesh; ^2^Hi-Tech Health Care Ltd., Banani, Dhaka-1213, Bangladesh

## Abstract

Human exposure to nonessential trace elements occurs from food crops that are contaminated by the soil. The present study aimed to determine the level of heavy metals in soil and rice samples using an atomic absorption spectrophotometer from three major industrial areas in Bangladesh: Savar, Gazipur, and Ashulia. Heavy metals were detected in the order Fe > Zn > Ni > Cr > Pb > Co > Cu > Cd > As and Zn > Cu > Cr > Co > Fe > Cd > Pb > Ni > As in the soil and rice samples, respectively. From this analysis, it was observed that the detected concentrations of Zn, Cd, Cr, and Co were higher than the WHO/FAO recommended maximum tolerance values. The transfer factor of the detected heavy metals from soil to rice was detected in the following order: Zn > Cu > Cr > Co > Cd > Pb > Fe > As > Ni. The accumulation of heavy metals in rice is a major public health concern. Therefore, we recommend strict regulations for the safety of food crops grown in the study areas.

## 1. Introduction

Heavy metals are toxic because they are persistent environmental pollutants [1, 2]. One major concern regarding heavy metals is that they are neither destroyed nor degraded, although their chemical forms may change. Heavy metal-mediated environmental contamination has become a global issue in recent years because of extensive industrialization worldwide [3]. Metal contamination in soil is the easiest way to expose humans to metals [2]. These heavy metals can accumulate in human tissues through the consumption of fruits, crops, and vegetables grown in contaminated soil [2, 3] and cause serious health risks [4]. Heavy metal accumulation in crops depends on several factors, including the type of plant, soil characteristics, selectivity to the crops, and permissibility of the metals [1, 3]. The term “accumulation factor” is used to indicate the heavy metal concentration in soil with respect to plants [5–8].

Exposure to metals through the digestion of contaminated food crops is a public concern because of the presence of heavy metals in the soils in industrial areas. Dietary intake is possibly the most important human exposure method, but inhalation is another exposure medium [4, 9]. Long-term exposure to heavy metals can cause serious health problems even when ingested at trace levels [9].

Essential metals, such as Cu and Zn, can bioaccumulate in animal and human bodies and may cause serious health problems when they reach their excessive limit [9, 10]. Several essential nutrients are removed from the human body after consumption of contaminated foods, which causes serious health issues [4]. Contamination of crops with heavy metals is a serious global issue [11]. In Bangladesh, industrialization is rapidly expanding; hence, environmental pollution is increasing [4]. Therefore, health risk assessment, calculated as the daily dietary intake of heavy metals from contaminated foods, is very important for both people.

There are many sources of heavy metal contaminations [12]. Several necessary nutrients, such as N, P, and K, are acquired by plants from the soil; unnecessary toxic metals can also be gathered from the soil as some plants can accumulate high heavy metal concentrations [13]. Heavy metals can be transferred from soil to crops through roots or shoots [14]. Toxic metals such as Pb, Cd, and As are transferred from the soil and stored in cereal grains [15]. The transfer capability of heavy metals from soil to crops affects their bioaccumulation pattern [16].

Rice plays an important role in the human diet [17], especially in Bangladesh. Rice provides vitamins, minerals, and amino acids to consumers worldwide [18]. The 2016 BBS reports [19] that approximately 80 different varieties of rice are cultivated in Bangladesh in three different seasons, and approximately 34.7 million metric tons of rice are produced per year. Approximately, 80% of Bangladeshi people consume rice three times a day [20]. Currently, the presence of toxic metals in arable lands and their transfer to crops, such as rice, are major concerns [21]. Crops grown in contaminated soil can accumulate large amounts of heavy metals in their tissues [22–24]. In Bangladesh, the accumulation of toxic metals in crops such as rice is a major concern [25]. The use of industrial wastes, agricultural chemicals, wastes from ship-breaking industries, and mining are the main sources of toxic metal contamination in the surrounding environment [26–29]. The presence of several toxic metals, such as Cd, Cu, Pb, and Hg in rice is a matter of great concern [30]. Three different studies showed an average lead content of 0.69 mg/kg in rice in southeast China [31], Cd content with maximum concentration of 0.467 mg/kg [21], and Pb content of 0.957 mg/kg [32]. These studies support the possibility of contamination of rice plants and grains by heavy metals. As rice, which is mostly contaminated by heavy metals, is the major food crop consumed in Bangladesh, there are possible carcinogenic and noncarcinogenic health risks.

The study area includes many textile, garment, tannery, pharmaceutical, and food industries. The major effluents from these industries are suspended solid; biological oxygen demand (BOD); chemical oxygen demand (COD); synthetic dyes; toxic chemicals; sulfur; alkalis; hydrogen peroxide; toxic heavy metals, including Cu, Cr, Cd, Zn, Ni, As, and Pb; dissolved oxygen; oil; grease; fats; sugar; color; preservatives; sulfides; ammonium salts; calcium salts; and nutrients, such as nitrogen, ammonia, and phosphates [33–36]. A large portion of these effluents is discharged directly into water streams that contaminate soil and crops. To date, no study has been conducted to detect the transfer of heavy metals into field crops in industrial areas, such as Savar, Gazipur, and Ashulia as the local inhabitants are regularly growing crops and consuming them. By detecting the metal contents present in crops in relation to soil, it is possible to assess the potential human health risks after their consumption.

The present study aimed to detect metal contamination levels in the soils of three major industrial areas of Bangladesh and to determine the metal concentrations, due to industrial activities, in food crops grown in those areas. Rice was the study's model crop because of its staple food status in Bangladesh. The findings of this study may provide insights into the metal accumulation factor of field crops from the soil and potential human health risks through the consumption of crops contaminated with heavy metals.

## 2. Methods and Materials

### 2.1. The Study Area

Soil and rice samples were collected from three main industrial areas in Bangladesh, Savar, Ashulia, and Gazipur. Savar is an Upazila of the Dhaka district that is located approximately 24 km northwest of Dhaka city. Savar is situated at 23°51′30″N 90°16′00″E/23.8583°N 90.2667°E/23.8583; 90.2667 with a total area of 280.13 km^2^. Savar is situated on the bank of the Banshi River. River water is used for drinking, bathing, and irrigation. However, because of industrialization and the huge dumping of industrial waste, the river water has become heavily polluted. Ashulia is a thana under Savar Upazila that is situated on the left bank of the Turag River. Ashulia is situated at 23° 53′ 59.1936″ N/90° 19′ 23.0952″ E, covering a total area of 27.186 km^2^. The Gazipur District is part of the Dhaka Division situated at 23° 59′ 59.7876″ 90° 25′ 12.9828″ E. The total area of Gazipur is approximately 1741.53 km^2^.

### 2.2. Sample Collection and Preparation for Heavy Metal Analysis

In each study area, soil samples were collected from several locations in the rice fields. At least three replicates of each sample were collected, leveled, and stored properly for further analysis. After collection, the soil samples were dried (50°C for 24 h), measured, ground to small powder using a mortar and pestle, and stored in a glass bottle for further analysis. The rice samples were collected, using the same sampling pattern, from the same field where the soil samples were collected. For rice sampling, a high-yielding rice variety from Bangladesh (BRRI Dhan 28) was collected. Samples were collected by hand, and cross-contamination was avoided. The samples were collected from at least ten locations in each sampling site between April and August 2021. Afterwards, they were dried at 70–80°C until a constant weight was reached [37].

### 2.3. Determination of Heavy Metal Concentrations in Soil and Rice Samples

The concentrations of heavy metals were detected in both soil and rice using an atomic absorption spectrophotometer (iCE-3000 series, Thermo Scientific, USA). An air-acetylene flame was used to ensure maximum sensitivity during the instrument operation. About 0.3 g of ground soil and rice grains were digested using a microwave digestion system (Berghof Speedwave, Germany) with 5 ml of 70% HNO_3_ and 2 ml of 30% H_2_O_2._ After digestion, Milli-Q water was added to the digested samples to make a final volume of 25 mL. The chemicals used for this analysis were of analytical grade and purchased from Merck (Germany). All the digested samples were then filtered using a 0.45 *µ*m filter syringe. Before analysis, all the consumables were soaked in diluted HNO_3_ for 24 h and finally rinsed with distilled water.

The limits of detection for Fe, Cu, Zn, Cd, Pb, Cr, Co, Ni, and As were 0.9, 0.8, 0.6, 0.07, 0.10, 0.8, 0.07, 0.8, and 0.09 ng/l, respectively. Certified reference materials (Sigma Aldrich, USA) were used to ensure the good precision of the applied method.

### 2.4. Analysis of Transfer Factor of Heavy Metals

The ability of plants to transfer metals from the soil can be determined through transfer factor analysis. The following formula was used for this analysis:

(1) $$\begin{array}{c} \text{TF}=\frac{C_{\text{Plant}}}{C_{\text{Soil}}}. \end{array}$$

Here, *C*_plant_ and *C*_soil_ represent the total metal concentration in the plant part (mg/kg) and soil (mg/kg) on a dry-weight basis, respectively [38].

### 2.5. Statistical Analysis

Analyses and extractions were performed in triplicate. Statistical analysis was performed using the Statistics 10 software.

## 3. Results and Discussion

### 3.1. Traces of Heavy Metals Detected in Soil Samples

The results of the detected heavy metals in the soil and rice samples from three different industrial areas are presented in Tables 1 and 2, respectively. The average Fe concentrations were 873.61 ± 112.09 mg/kg, 668.34 ± 98.06 mg/kg, and 976.12 ± 32.45 mg/kg in the soil samples of Savar, Gazipur, and Ashulia, respectively. Higher Fe concentrations were detected in the soil samples from Ashulia, followed by those of Savar and Gazipur. The average Cu contents were 31.54 ± 7.23 mg/kg, 19.76 ± 5.97 mg/kg, and 29.65 ± 7.34mg/kg in the soil samples of Savar, Gazipur, and Ashulia, respectively. The Cu concentrations of most soil samples (47%) were within the range of the average shale value (45 mg/kg), indicating contamination at the sampling sites. The Cu concentrations detected in the sampling areas were similar to those of a previous study [39]. The present study indicated that the Cu concentrations were lower compared to those of other industrial cities in the world [40].

**Figure 1:**
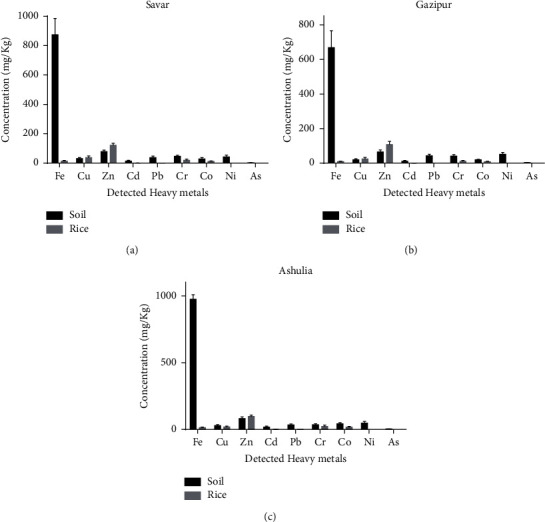
Comparative heavy metal concentration in soil and rice. (a) Concentrations of heavy metals in the Savar area; (b) concentrations of heavy metals in the Gazipur area; and (c) concentrations of heavy metals in the Ashulia area.

The detected heavy metals indicated contamination due to human activities in the industrial areas. Toxic metals from wastewater are mixed with soil and finally transferred to crops, resulting in serious health threats for both humans and animals. The average Zn values were 78.65 ±10.54 mg/kg, 64.98 ± 11.89 mg/kg, and 81.90 ± 12.87 mg/kg in the soils of Savar, Gazipur, and Ashulia, respectively. The comparative heavy metal concentrations in soil and rice are presented in Figure 1. This study reported that approximately 68% of the soil samples contained higher Zn values than the average shale value (0.095 mg/kg). The Zn concentrations in the soil samples of the study areas were higher than those in other major industrial cities of the world [40]. A study reported that the Zn concentrations in river alluvium soils were 78.50 mg/kg and 66.4 mg/kg, respectively [41]. Other heavy metals, including Cd, Pb, Cr, Co, and Ni, were detected in the soil samples from Savar, Gazipur, and Ashulia. The following were detected: Cd (11.08 ± 4.98 mg/kg–18.56 ± 6.75 mg/kg), Pb (34.09 ± 7.90 mg/kg–42.78 ±8.54 mg/kg), Cr (34.87 ± 8.74 mg/kg–46.93 ± 7.54 mg/kg), Co (19.56 ± 3.54 mg/kg–43.09 ± 8.69 mg/kg), Ni (43.25 ±12.53 mg/kg–51.76 ± 10.65 mg/kg), and As in small concentration (2.98 ± 1.12 mg/kg–3.76 ± 1.54 mg/kg).

The average Fe concentrations in rice samples were 14.89 ± 3.45 mg/kg, 9.49 ± 2.34 mg/kg, and 11.87 ± 3.34 mg/kg in Savar, Gazipur, and Ashulia, respectively. Similarly, the average Cu concentrations were 38.12 ± 11.21 mg/kg, 25.34 ± 8.56 mg/kg, and 19.74 ± 5.87 mg/kg, and average Zn concentrations were 121.76 ± 13.98 mg/kg, 107.43 ±18.54 mg/kg, and 97.34 ± 10.73 mg/kg in the soil samples of Savar, Gazipur, and Ashulia, respectively. Other heavy metals, including Cd, Pb, Cr, Co, Ni, and As, were also detected in the soil samples. The following were detected: Cd (0.98 ± 0.32 mg/kg–1.61 ± 0.79 mg/kg), Pb (ND–1.32 mg/kg), Cr (11.54 ± 4.09–23.67 ± 9.95 mg/kg), Co (8.54 ± 3.32–18.11 ± 5.09 mg/kg), Ni (ND–0.18 mg/kg), and As (0.031 ± 0.01–0.075 ± 0.03 mg/kg) in the crop samples of Savar, Gazipur, and Ashulia, respectively.

### 3.2. Transfer Factor (TF) of Toxic Metals from Soil to Rice

**Figure 2:**
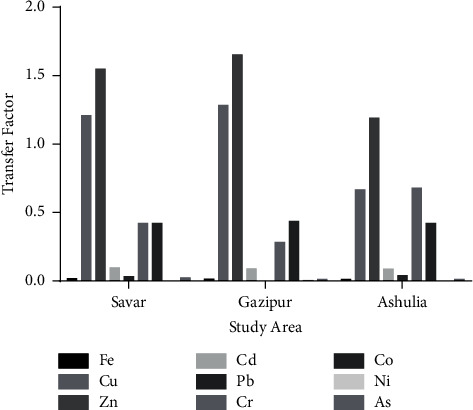
Comparative transfer factor (TF) of heavy metals in Savar, Gazipur, and Ashulia.

The transfer factor (TF) is defined as the capability of plants to absorb ionic metals through their roots to aerial parts [42]. The presence of heavy metals in plant tissues in relation to the soil's heavy metal concentrations can be determined using transfer factor analysis. The TF is an important term for determining the transfer efficiency of heavy metals. Metals with high TF are easily transferred to crops unlike metals with low TF. The TFs of heavy metals from the soil to crops are presented in Table 3. Variances in heavy metal content may depend on their concentrations in soil and plant parts [43]. The present study showed that higher amounts of Cu and Zn were transferred from the soil to rice than other metals. The average TF values of Cu were 1.21, 1.28, and 0.67, whereas the average TF values of Zn were 1.55, 1.65, and 1.19 in Gazipur and Ashulia, respectively. Ni, As, and Pb showed lower TF values than the other heavy metals. The average TF values of Ni were 0, 0.003, and 0; the average TF values of As were 0.02, 0.01, and 0.01; and the average TF values of Pb were 0.03, 0, and 0.03 for rice samples of Savar, Gazipur, and Ashulia, respectively. The comparative transfer factors of Savar, Gazipur, and Ashulia are shown in Figure 2.

The ability of Ni, As, and Pb to form stable complexes with amino acids [44] may be the reason for their low TFs. Other factors, such as soil pH and soil properties, also influence metal TFs from soil to crops [45, 46].

The Savar, Gazipur, and Ashulia regions are at high risk because of environmental pollution due to rapid industrialization, huge population, and urbanization in the last 20 years. This study investigated the levels of selected heavy metals in soil and rice from the agricultural lands of the Savar, Gazipur, and Ashulia industrial areas. The study aimed to focus on the contamination status of soil and rice and to identify the interactions between soil and rice metal concentrations. Although Savar, Gazipur, and Ashulia are industrial areas, farmers produce different crops throughout the year and supply them locally and nationwide. Soil contamination with heavy metals results in crop contamination, which is a major health issue.

Because of rapid industrialization nationwide, soil, air, and water are becoming increasingly polluted because of the inadequate disposal of waste materials to the environment. Crops are also becoming increasingly polluted as a result of soil pollution. Savar, Gazipur, and Ashulia are three major industrial areas of the Dhaka Division, and significant amounts of heavy metals are discharged into the soil, which is finally transferred to crops and accumulated in edible grains. Heavy metals have been detected at high concentrations in crops grown in industrial areas of Bangladesh [2–4, 8].

Heavy metal pollution is one of the biggest problems of not only developing countries, such as Bangladesh, but also worldwide [3]. The effect of wastewater on crops has led to a change in the soil physiochemical nature (such as pH), which has a significant influence on heavy metal mobility and bioavailability in crops [47]. Continuous wastewater irrigation results in elevated levels of heavy metals in soil and food crops. In general, the transfer of heavy metals from the soil to plants is a key component of human exposure to these metals [48]. Rice is a staple food in Bangladesh and an important component of the human diet. The intake of heavy metal-contaminated rice causes several health problems, such as DNA damage, that may reduce energy levels. At the molecular level, heavy metals interact with the thiol, amino, and imino groups of proteins to form metal complexes, thus inhibiting protein activities [49]. Humans encounter heavy metals through the intake of contaminated foods, polluted air inhalation, or exposure in their daily lives [50]. The transfer route is from industry to the environment (soil and water), foods (crops and vegetables), and finally humans [51]. Heavy metals such as Pb, Cd, Mn, and As can enter the body through the mouth and gastrointestinal system during food ingestion, while other heavy metals can enter the body through inhalation. Lead can be absorbed through the skin.

Several human health effects can be observed owing to heavy metal toxicity. The functions of the brain, kidneys, lungs, liver, and blood can be altered by heavy metal toxicity [52]. Several heavy metals, including Fe, Cu, Cr, Co, and As, generate free radicals and induce oxidative stress and oxidation of biological molecules [53]. Certain heavy metals, such as Pb, Hg, Ni, Cd, and Fe, have carcinogenic effects. Heavy metals can target signaling and cellular regulatory proteins that are responsible for apoptosis, regulation of the cell cycle, DNA repair, DNA methylation, and cell growth and differentiation [54]. Heavy metals such as Pb and Mn can also induce neurotoxicity [55]. Heavy metals can also affect several plant functions, including nitrogen fixation, chlorosis, and plant growth and metabolism [49]. Several minerals, nutrients, and organic and inorganic matter are stored in the soil [3, 49]. Soil is polluted by heavy metals through natural, industrial, and human activities [4, 56–59]. Heavy metal exposure through food may cause serious health problems, including various fetal diseases.

In Bangladesh, several studies have been performed to detect heavy metal content in soil [3, 40, 60–62] and crops [41, 63–66]. A study reported that the concentrations of heavy metals, such as Cu and Pb, are higher than the permissible values in Zirani and Savar [67]. Similarly, another study detected higher concentrations of heavy metals such as Mn, Zn, Fe, and Cu than the standard regulatory limit in Dhaka [68]. In Khulna, the soil is contaminated with Pb and Cd [69]. Another study reported that the soil of Bogura city was contaminated with Cu and Cd [70]. The toxicity of heavy metals begins when they accumulate in soft tissues after ingestion [71]. When heavy metals are regularly taken above the acceptable daily limit through food, they become harmful to human health. The harmful effects of Cd, Pb, As, Zn, and Cu have also been reported [72]. Low Pb concentrations may lead to developmental defects in children [73], whereas high levels (75 *μ*g/dL) may lead to coma and even death. Cd is considered a neurotoxicant in several animal models [74]. Ni is responsible for several disorders, including chronic bronchitis, emphysema, impaired pulmonary function, and fibrosis [75]. Excessive intake of Cu and Cr may be toxic [76]; although Cr helps to maintain the blood glucose level, Cr is used as a medication for diabetes [77].

In the present study, heavy metals such as Fe, Cu, Zn, Cr, Co, Ni, Pb, Cd, and As were detected in the soil samples, whereas higher amounts of Cu, Zn, Fe, Cr, and Co were detected in rice. From the analysis, it was observed that the concentrations of Zn, Cd, Cr, and Co were higher than the WHO/FAO recommended maximum tolerance values [78]. Therefore, the consumption of rice grown in these industrial areas is a major concern and regular monitoring is strongly recommended.

## 4. Conclusion

The results of this study revealed the presence of Fe, Zn, Cu, Pb, Cr, and Co, Ni, and As in soil and rice samples from three major industrial areas of the Dhaka division in Bangladesh. The average concentrations of Fe, Cu, and Zn were higher than those of Pb, Cr, Co, Ni, and As, indicating that the former are the major contaminants in these industrial areas. Food consumption is one of the major routes of exposure to heavy metals in humans. The possible transfer rate of these toxic heavy metals from contaminated soil to rice is also reported in the present study. The presence of hazardous heavy metals in food crops, such as rice, may lead to serious health problems. This study was based on selected spots of major industrial areas, and the background values of different heavy metals were different; therefore, the presence of their contents in the environment does not represent the proper pollution level in a particular area. However, environmental protection laws should be properly maintained to reduce environmental pollution, and more attention should be paid to minimizing contamination in the studied areas.

## Figures and Tables

**Table 1:** Average heavy metal concentration in soil samples of Savar, Gazipur, and Ashulia.

Heavy metals (mg/kg)	Fe	Cu	Zn	Cd	Pb	Cr	Co	Ni	As	
Soil	873.61	31.54	78.65	14.98	38.07	46.93	28.65	43.25	3.32	Savar
	668.34	19.76	64.98	11.08	42.78	40.76	19.56	51.76	2.98	Gazipur
	976.12	29.65	81.9	18.56	34.09	34.87	43.09	47.96	3.76	Ashulia

**Table 2:** Average heavy metal concentration in rice samples of Savar, Gazipur and Ashulia.

Heavy metals (mg/kg)	Fe	Cu	Zn	Cd	Pb	Cr	Co	Ni	As	
Rice	14.89	38.12	121.76	1.43	1.21	19.78	12.08	ND	0.075	Savar
	9.49	25.34	107.43	0.98	ND	11.54	8.54	0.18	0.031	Gazipur
	11.87	19.74	97.34	1.61	1.32	23.67	18.11	ND	0.048	Ashulia

WHO/FAO recommended maximum tolerance value (mg/kg)	450	40	60	0.3	5	5	0.2	0.1	0.5	

^*∗*^ND: not detected.

**Table 3:** Transfer factor (TF) of heavy metals in rice from Savar, Gazipur, and Ashulia.

Transfer factor	Fe	Cu	Zn	Cd	Pb	Cr	Co	Ni	As
Savar	0.01704	1.20862	1.548125	0.09546	0.0318	0.421479	0.42164	0	0.02259
Gazipur	0.0142	1.28239	1.653278	0.08845	0	0.283121	0.436605	0.00348	0.0104
Ashulia	0.01216	0.66577	1.188523	0.08675	0.0387	0.678807	0.420283	0	0.01277

## Data Availability

All the data used in this manuscript are available from the corresponding author upon request.

## References

[1] Aktaruzzaman M., Chowdhury M. A. Z., Fardous Z., Alam M. K., Hossain M. S., Fakhruddin A. N. M. (2014). Ecological risk posed by heavy metals contamination of ship breaking yards in Bangladesh. *International Journal of Environmental Research*.

[2] Chowdhury N., Rasid M. M. (2016). Heavy metal contamination of soil and vegetation in ambient locality of ship breaking yards in Chittagong, Bangladesh. *Journal of Environmental Science, Toxicology and Food Technology*.

[3] Ahmad J. U., Goni M. A. (2010). Heavy metal contamination in water, soil, and vegetables of the industrial areas in Dhaka, Bangladesh. *Environmental Monitoring and Assessment*.

[4] Zhuang P., McBride M. B., Xia H., Li N., Li Z. (2009). Health risk from heavy metals via consumption of food crops in the vicinity of Dabaoshan mine, south China. *The Science of the Total Environment*.

[5] Wang C., Ji J., Chen M., Zhong C., Yang Z., Browne P. (2017). Atmospheric contribution to boron enrichment in aboveground wheat tissues. *Chemosphere*.

[6] Wang C., Ji J., Zhu F. (2017). Characterizing se transfer in the soilcrop systems under field condition. *Plant and Soil*.

[7] Selinus O., Centeno J. A., Finkelman R. B., Fuge R., Lindh U., Smedley P. (2005). *Essentials of Medical Geology: Impacts of the Natural Environment on Public Health*.

[8] Miclean M., Cadar O., Levei E. A., Roman R., Ozunu A., Levei L. (2019). Metal (Pb, Cu, Cd, and Zn) transfer along food chain and health risk assessment through raw milk consumption from free-range cows. *International Journal of Environmental Research and Public Health*.

[9] Cadar O., Miclean M., Cadar S., Tanaselia C., Senila L., Senila M. (2015). Assessment of heavy metals in cows milk in Rodnei mountains area, Romania. *Environmental Engineering and Management Journal*.

[10] Chandra K. G., Pandey P., Singh N. M. P., Mishra V. (2011). Uptake and accumulation of potentially toxic metals (Zn, Cu and Pb) in soils and plants of Durgapur industrial belt. *Journal of Environmental Biology*.

[11] Miclean M., Cadar O., Levei L., Senila L., Ozunu A. (2018). Metal contents and potential health risk assessment of crops grown in a former mining district (Romania). *Journal of Environmental Science and Health, Part B*.

[12] Fabjola B., Marco L., Laura B. (2015). Evaluation of heavy metals contamination from environment to food matrix by TXRF: the case of rice and rice husk. *Journal of Chemistry*.

[13] Watanabe M. E. (1997). Phytoremediation on the brink of commercialization. *Environmental Science & Technology*.

[14] Uchida S., Tagami K., Ishikawa N. Concentration, soil-to-plant transfer factor and soil-soil solution distribution coefficient of selentum in the surface environment (9106).

[15] Hu X.-F., Jiang Y., Shu Y., Hu X., Liu L., Luo F. (2014). Effects of mining wastewater discharges on heavy metal pollution and soil enzyme activity of the paddy fields. *Journal of Geochemical Exploration*.

[16] Sekara A., Poniedzialek M., Ciura J., Jedrszczyk E. (2005). Cadmium and lead accumulation and distribution in the organs of nine crops: implications for phytoremediation. *Polish Journal of Environmental Studies*.

[17] Proshad R., Kormoker T., Islam M. S., Chandra K. (2019a). Potential health risk of heavy metals via consumption of rice and vegetables grown in the industrial areas of Bangladesh. *Human and Ecological Risk Assessment: An International Journal*.

[18] Zeng Y., Wang L., Du J. (2009). Elemental content in brown rice by inductively coupled plasma atomic emission spectroscopy reveals the evolution of Asian cultivated rice. *Journal of Integrative Plant Biology*.

[19] BBS (2016). *Yearbook of Agricultural Statistics-2015, Bangladesh: Bangladesh Bureau of Statistics; Statistics and Informatics Division*.

[20] Islam M. S., Ahmed M. K., Habibullah-Al-Mamun M., Raknuzzaman M. (2015). The concentration, source and potential human health risk of heavy metals in the commonly consumed foods in Bangladesh. *Ecotoxicology and Environmental Safety*.

[21] Zhao K., Liu X., Xu J., Selim H. M. (2010). Heavy metal contaminations in a soil-rice system: identification of spatial dependence in relation to soil properties of paddy fields. *Journal of Hazardous Materials*.

[22] Sharma R. K., Agrawal M., Marshall F. (2006). Heavy metal contamination in vegetables grown in wastewater irrigated areas of Varanasi, India. *Bulletin of Environmental Contamination and Toxicology*.

[23] Kumar Sharma R., Agrawal M., Marshall F. (2007). Heavy metal contamination of soil and vegetables in suburban areas of Varanasi, India. *Ecotoxicology and Environmental Safety*.

[24] Marshall F. M., Holden J., Ghose C. (2007). Contaminated irrigation water and food safety for the urban and peri-urban poor: appropriate measures for monitoring and control from field research in India and Zambia. *Incpetion Rep DFID Enkar*.

[25] Bishwajit G., Barmon R., Ghosh S. (2014). Reviewing the status of agricultural production in Bangladesh from a food security perspective. *Russian Journal of Agricultural and Socio-Economic Sciences*.

[26] Granero S., Domingo J. L. (2002). Levels of metals in soils of Alcalá de Henares, Spain. *Environment International*.

[27] Han W.-Y., Zhao F.-J., Shi Y.-Z., Ma L.-F., Ruan J.-Y. (2006). Scale and causes of lead contamination in Chinese tea. *Environmental Pollution*.

[28] Lee J.-S., Chon H.-T., Kim K.-W. (2005). Human risk assessment of As, Cd, Cu and Zn in the abandoned metal mine site. *Environmental Geochemistry and Health*.

[29] Kamani H., Mirzaei N., Ghaderpoori M., Bazrafshan E., Rezaei S., Mahvi A. H. (2018). Concentration and ecological risk of heavy metal in street dusts of Eslamshahr, Iran. *Human and Ecological Risk Assessment: An International Journal*.

[30] McLaughlin M. J., Singh B. R. (1999). Cadmium in soils and plants. *Cadmium in Soils and Plants*.

[31] Fu J., Zhou Q., Liu J. (2008). High levels of heavy metals in rice (*Oryza sativa* L.) from a typical E-waste recycling area in southeast China and its potential risk to human health. *Chemosphere*.

[32] Hang X., Wang H., Zhou J., Ma C., Du C., Chen X. (2009). Risk assessment of potentially toxic element pollution in soils and rice (*Oryza sativa*) in a typical area of the Yangtze river delta. *Environmental Pollution*.

[33] Dey S., Islam A. (2015). A review on textile wastewater characterization in Bangladesh. *Resources and Environment*.

[34] Kamal A. K. I., Ahmed F., Hassan M., Uddin M., Hossain S. M. (2016). Characterization of textile effluents from Dhaka export processing zone (DEPZ) area in Dhaka, Bangladesh. *Pollution*.

[35] Chowdhury M., Mostafa M. G., Biswas T. K., Mandal A., Saha A. K. (2015). Characterization of the effluents from leather processing industries. *Environmental Processes*.

[36] Kumari V., Tripathi A. K. (2019). Characterization of pharmaceuticals industrial effluent using GC–MS and FT-IR analyses and defining its toxicity. *Applied Water Science*.

[37] Tiwari K. K., Singh N. K., Patel M. P., Tiwari M. R., Rai U. N. (2011). Metal contamination of soil and translocation in vegetables growing under industrial wastewater irrigated agricultural field of Vadodara, Gujarat, India. *Ecotoxicology and Environmental Safety*.

[38] Li Q., Chen Y., Fu H. (2012). Health risk of heavy metals in food crops grown on reclaimed tidal flat soil in the Pearl river estuary, China. *Journal of Hazardous Materials*.

[39] Domingo L. E., Kyuma K. (1983). Trace elements in tropical Asian paddy soils. *Soil Science & Plant Nutrition*.

[40] Zakir H. M., Sumi S. A., Sharmin S., Mohiuddin K. M., Kaysar S. (2015). Heavy metal contamination in surface soils of some industrial areas of Gazipur, Bangladesh. *Journal of Chemical, Biological and Physical Sciences*.

[41] Jahiruddin M., Harada H., Hatanaka T., Islam M. R. (2000). Status of trace elements in agricultural soils of Bangladesh and relationship with soil properties. *Soil Science & Plant Nutrition*.

[42] Olguín E. J., Sánchez-Galván G. (2012). Heavy metal removal in phytofiltration and phycoremediation: the need to differentiate between bioadsorption and bioaccumulation. *New Biotech*.

[43] Millaleo R., Reyes- Diaz M., Ivanov A. G., Mora M. L., Alberdi M. (2010). Manganese as essential and toxic element for plants: transport, accumulation and resistance mechanisms. *Journal of Soil Science and Plant Nutrition*.

[44] Mengel K., Kirkby E. A., Kosegarten H., Appel T. (2001). Nitrogen. *Principles of Plant Nutrition*.

[45] Li X., Zhou Q., Wei S., Ren W., Sun X. (2011). Adsorption and desorption of carbendazim and cadmium in typical soils in northeastern China as affected by temperature. *Geoderma*.

[46] Islam M. S., Ahmed M. K., Habibullah-Al-Mamun M. (2016). Apportionment of heavy metals in soil and vegetables and associated health risks assessment. *Stochastic Environmental Research and Risk Assessment*.

[47] Nigam R., Srivastava S., Prakash S., Srivastava M. M. (2001). Cadmium mobilization and plant availability-the impact of organic acids commonly exuded from roots. *Plant and Soil*.

[48] Khan S., Cao Q., Zheng Y. M., Huang Y. Z., Zhu Y. G. (2008). Health risks of heavy metals in contaminated soils and food crops irrigated with wastewater in Beijing, China. *Environmental Pollution*.

[49] Kumar A., Seema (2016). Accumulation of heavy metals in soil and green leafy vegetables, irrigated with wastewater. *Journal of Environmental Science, Toxicology and Food Technology*.

[50] Yu M. H., Tsunoda H. (2004). *Environmental Toxicology: Biological and Health Effects of Pollutants*.

[51] Keshav Krishna A., Rama Mohan K. (2016). Distribution, correlation, ecological and health risk assessment of heavy metal contamination in surface soils around an industrial area, Hyderabad, India. *Environmental Earth Sciences*.

[52] Engwa G. A., Ferdinand P. U., Nwalo F. N., Unachukwu M. N. (2019). Mechanism and health effects of heavy metal toxicity in humans. *Poisoning in the Modern World-New Tricks for an Old Dog*.

[53] Valko M., Morris H., Cronin M. (2005). Metals, toxicity and oxidative stress. *Current Medicinal Chemistry*.

[54] Kim H. S., Kim Y. J., Seo Y. R. (2015). An overview of carcinogenic heavy metal: molecular toxicity mechanism and prevention. *Journal of Cancer Prevention*.

[55] Neal A. P., Guilarte T. R. (2012). Mechanisms of heavy metal neurotoxicity: lead and manganese. *Journal of Drug Metabolism & Toxicology*.

[56] Zheng N., Liu J., Wang Q., Liang Z. (2010). Health risk assessment of heavy metal exposure to street dust in the zinc smelting district, northeast of China. *The Science of the Total Environment*.

[57] Calderón J., Ortiz-Pérez D., Yáñez L., Díaz-Barriga F. (2003). Human exposure to metals: pathways of exposure, biomarkers of effect, and host factors. *Ecotoxicology and Environmental Safety*.

[58] Wang X. L., Sato T., Xing B. S., Tao S. (2005). Health risks of heavy metals to the general public in Tianjin, China via consumption of vegetables and fish. *The Science of the Total Environment*.

[59] Bouchard M. F., Sauvé S., Barbeau B. (2011). Intellectual impairment in school-age children exposed to manganese from drinking water. *Environmental Health Perspectives*.

[60] Zaman S., Rajonee A. A., Huq S. M. I. (2017). Arsenic in Bangladesh soils and its relationship with water soluble soil organic carbon. *Open Journal of Soil Science*.

[61] Islam S. M., Tusher T. R., Mustafa M., Mamun S. A. (2012). Investigation of soil quality and heavy metal concentrations from a waste dumping site of Konabari industrial area at Gazipur in Bangladesh. *IOSR Journal of Environmental Science, Toxicology and Food Technology*.

[62] Ahmed F., Fakhruddin A. N. M., Imam M. T. (2016). Spatial distribution and source identification of heavy metal pollution in roadside surface soil: a study of Dhaka Aricha highway, Bangladesh. *Ecological Processes*.

[63] Jolly Y. N., Huque R., Islam A. (2014). Toxic elements in rice and possible health risk assessment–Bangladesh prospect. *Journal of Food Process*.

[64] Kormoker T., Proshad R., Islam M. S. (2020). Presence of toxic metals in rice with human health hazards in Tangail district of Bangladesh. *International Journal of Environmental Health Research*.

[65] Yasmin F., Simol H. A., Sultana G. N. N. (2019). Heavy and trace elements in some varieties of rice consumed in Dhaka city of Bangladesh. *International Journal of Sciences*.

[66] Uddin M. N., Hasan M. K., Dhar P. K. (2019). Contamination status of heavy metals in vegetables and soil in Satkhira, Bangladesh. *Journal of Materials and Environmental Science*.

[67] Nessa F., Jewel A. H. (2014). Analysis of soil nutrient and heavy metal concentration in agricultural land of Zirani industrial area, Savar, Dhaka. *International Journal of Innovation and Scientific Research*.

[68] Hasnine M. T., Huda M. E., Khatun R. (2017). Heavy metal contamination in agricultural soil at DEPZA, Bangladesh. *Environment and Ecology Research*.

[69] Fahmida K., Rafizul I. M. (2017). An investigation on soil quality and heavy metal levels in soil of rajbandh waste disposal site at Khulna, Bangladesh. *Iranian Journal of Energy and Environment*.

[70] Begum K., Mohiuddin K. M., Zakir H. M., Rahman M. M., Hasan M. N. (2014). Heavy metal pollution and major nutrient elements assessment in the soils of Bogra city in Bangladesh. *Canadian Chemical Transactions*.

[71] Sobha K., Poornima A., Harini P., Veeraiah K. (2007). A study on biochemical changes in the fresh water fish, catla catla (hamilton) exposed to the heavy metal toxicant cadmium chloride. *Kathmandu University Journal of Science, Engineering and Technology*.

[72] Jaishankar M., Tseten T., Anbalagan N., Mathew B. B., Beeregowda K. N. (2014). Toxicity, mechanism and health effects of some heavy metals. *Interdisciplinary Toxicology*.

[73] Ihedioha J. N., Ujam O. T., Nwuche C. O., Ekere N. R., Chime C. C. (2016). Assessment of heavy metal contamina¬tion of rice grains (*Oryza sativa*) and soil from ada field, Enugu, Nigeria: estimating the human health risk. *Human and Ecological Risk Assessment: An International Journal*.

[74] United States Department of Health and Human Services (2007). *Toxicological Profile for Lead*.

[75] United States Department of Health and Human Services (2005). *Agency for Toxic Substances and Disease Registry*.

[76] McDowell L. R. (2003). *Minerals in Animal and Human Nutrition*.

[77] Broadhurst C. L., Domenico P. (2008). Clinical studies on chromium picolinate supplementation in diabetes mellitus—a review. *Diabetes Technology & Therapeutics*.

[78] Codex Alimentarious Commission (1984). Contaminants. *Joint FAO/WHO Food standards Program*.

